# Isolation and identification of *Streptococcus pneumoniae* serotype 6B from a patient with bacterial meningitis infection in Jakarta, Indonesia

**DOI:** 10.1099/acmi.0.000123

**Published:** 2020-03-26

**Authors:** Diana Shintawati Purwanto, Tonny Loho, Wisnu Tafroji, Irawan Mangunatmadja, Suzanna Immanuel, Ina Susianti Timan, Yusra Yusra, Dodi Safari

**Affiliations:** ^1^​ Department of Clinical Pathology, Faculty of Medicine Universitas Indonesia/Dr. Cipto Mangunkusumo Hospital, Jakarta, Indonesia; ^2^​ Department of Biochemistry, Faculty of Medicine Sam Ratulangi University, Manado, Indonesia; ^3^​ Molecular Bacteriology Unit, Eijkman Institute for Molecular Biology, Jakarta, Indonesia; ^4^​ Department of Child Health, Faculty of Medicine Universitas Indonesia/Dr. Cipto Mangunkusumo Hospital, Jakarta, Indonesia

**Keywords:** *Streptococcus pneumoniae*, meningitis, CSF infection, serotype 6B, antibiotic susceptibility

## Abstract

CNS infection is a life-threatening condition in developing countries and *Streptococcus pneumoniae* has been reported as the most common cause of bacterial meningitis; however, there is limited data on pneumococcal meningitis in Indonesia. This cross-sectional study aimed to isolate and identity *S. pneumoniae* strains from cerebrospinal fluid (CSF) specimens collected as part of routine testing from patients with clinically diagnosed central nervous system infection at a national referral hospital in Jakarta, Indonesia in 2017. *S. pneumoniae* isolation and identification were performed using conventional culture and molecular tools. Antibiotic susceptibility patterns were monitored through minimum inhibitory concentration testing. From 147 CSF specimens, one *S. pneumoniae* strain was identified from a patient with bacterial meningitis symptoms. The isolate was serotype 6B (ST5661) and susceptible to 18 antimicrobial agents tested, including penicillin, tetracycline, and the macrolide group. Our data provide insights into the epidemiology of invasive pneumococcal disease in Indonesia.

Bacteria, amoebae, fungi, and viruses are capable of central nervous system (CNS) invasion causing significant morbidity and mortality [1]. The incidence and etiology of CNS infections vary with time, by geographic region, with age, co-morbidities and vaccination policies [2]. It is a life-threatening condition in developing countries where *S. pneumoniae* has reported as the most common cause of bacterial meningitis [1–4]. Jayaraman, Y. *et al*. reported that *S. pneumoniae* serotypes causing bacterial meningitis in hospitalized children under five years of age in India were serotypes 6B, 14, 6A, and 19F which is included in pneumococcal vaccine (PCV) [5]. It was reported that introduction of the pneumococcal conjugate vaccines broadly reduced vaccine-type meningitis in different regions [6–8]. Currently, there is very limited data available on pneumococcal meningitis including pneumococcus serotype causing pneumococcal meningitis and its antimicrobial susceptibility profile in Indonesia. Recently, Imran *et al*. reported that tuberculosis, toxoplasmosis, and cryptococcosis are common causes of CNS infections in adult patients in a referral hospital in Jakarta, Indonesia [9]. Therefore, this study aimed to isolate, identify and determine the serotype of *S. pneumoniae* strains in cerebrospinal fluid (CSF) from patients clinically diagnosed with CNS infection at a national referral hospital, Jakarta, Indonesia.

This cross-sectional study was approved by the Institutional Review Board of Faculty of Medicine, Universitas Indonesia, Jakarta, Indonesia. A total of 147 CSF specimens from patients who were diagnosed with CNS infection were collected as a part of a routine testing at a national referral hospital, Jakarta from July to December 2017 (Table 1). These CSF specimens were centrifuged for 15 min at 1000 ***g*** and the supernatant was removed. This step was performed sequentially for each 1000 µl of CSF specimen [10, 11]. Direct Gram-staining was performed on this suspension, and the suspension was also cultured onto 5 % sheep blood agar followed by incubation at 37 °C and 5 % CO_2_ for 18–20 h. The plate was re-examined after 48 h of incubation [11]. The alpha-hemolytic colonies growing on the blood agar plate were sub-cultured, followed by optochin susceptibility testing, bile solubility testing, and Gram-staining [12], using *S. pneumoniae* ATCC 49619 as positive control for the testing. The remaining CSF specimens were stored at −80 °C until further processing. Matrix-assisted laser desorption ionization–time of flight mass spectrometry (MALDI-TOF MS) was used to confirm the bacterial identification [13, 14].

**Table 1.** Characteristics of patients with CNS infection in Jakarta, Indonesia

Variable	n (%)
Age (year)	
0–5	26 (18)
6–17	14 (10)
18–59	100 (68)
≥60	7 (5)
Sex	
Male	85 (58)
Source	
Ward	91 (62)
Emergency department	27 (18)
Pediatric intensive care unit	5 (3)
Polyclinic	4 (3)
Other hospitals	13 (9)
Others	24 (16)
Diagnosis/clinical information	
Tuberculous meningitis	28 (19)
Bacterial meningitis	24 (16)
Intracranial infection	21 (14)
Encephalitis	18 (l2)
Guillain Barre syndrome	17 (12)
Meningoensephalitis	6 (4)
Transverse myelitis	6 (4)
Others	27 (18)
Symptoms	
Fever	61 (42)
Headache	45 (31)
Extremity weakness	19 (13)
Nausea/vomiting	13 (9)
Alteration of mental status	6 (4)
Others	27 (18)
Signs	
Paresis/hemiparesis	40 (27)
Seizures	36 (25)
Decreased consciousness	31 (21)
Motor neuron weakness	23 (16)
Paresis of nervus facialis	19 (13)
Nuchal rigidity	10 (7)
Kernig and Brudzinski signs	8 (6)
Antibiotic use prior to lumbal puncture	
Yes	88 (60)
No	46 (31)
No data	13 (9)

Bacterial DNA was extracted as described previously [15]. The qPCR targeting the pneumococcal autolysin, encoded by *lytA* gene, was performed by using the Invitrogen Platinum Master Mix (Invitrogen, USA) [16]. Serotype was determined using sequential multiplex PCR [17] and confirmed through latex agglutination and Quellung antiserum reaction. Multilocus sequence typing was performed as described by Enrigh and Spratt [18]. Allele profiles and sequence type (ST) of the isolate was determined from the MLST database (http://www.pubmlst.org). Antibiotic susceptibility testing was performed by using the microdilution MIC Plate for *S. Pneumoniae* (Sensititre Thermoscientific) containing 20 antibiotics in a 96-well round bottom plate. This test was performed using an overnight culture to prepare the suspension in Mueller-Hinton broth equal to 0.5 McFarland. The suspension (100 µl) was transferred into 11 ml of lysed-horse blood and vortexed; next 100 µl of the mixture was transferred into each well. The MIC plate was incubated at 37 °C incubator for 18–20 h and examined using a mirror to observe the first clear well defined as the MIC value, which was interpreted using Clinical and Laboratory Standard Institute (CLSI 28th Edition) breakpoints for defining sensitivity.

The patient characteristics are described in Table 1. A total of 147 CSF specimens were collected from patients with clinically diagnosed with CNS infection, aged 2 months to 77.5 years; 58 % of the patients were male. The majority of the specimens were collected from the ward (62%), followed by the emergency department (18%) and other hospitals (9%) (Table 1). The clinical diagnoses of patients were tuberculous meningitis (19%), bacterial meningitis (16%), intracranial infection (14%), encephalitis (12%), and Guillain Barre syndrome (12%). General patient symptoms included fever (42%), headache (31%), and extremity weakness (13%), and specific CNS infection symptoms comprised paresis/hemiparesis (27%), seizures (25%), decreased consciousness (21%), motor neuron weakness (16%), paresis of nervus facialis (13%), nuchal rigidity (7%), and Kernig and Brudzinski signs (6%). We observed that most (60%) of the patients received antibiotic treatments prior to CSF specimen collection (Table 1).

One specimen (Specimen ID: 371016106) displayed Gram-positive lanceolate-shaped diplococci following direct Gram-staining (Fig. 1A), and alpha-hemolytic nonmucoid small translucent colonies appeared on blood agar after 24 h of incubation. Gram-staining of these colonies also showed Gram-positive lanceolate-shaped diplococci (Fig. 1B), and bile and optochin susceptibility tests identified the specimen as *S. pneumoniae *. Furthermore, this isolate was determined as serotype 6B and the sequence type (ST) of this strain was identified as ST5661. This isolate was susceptible to the following 18 antibiotics: moxifloxacin, levofloxacin, tetracycline, cefuroxime, ceftriaxone, chloramphenicol, penicillin, meropenem, ertapenem, amoxicillin/clavulanic acid (2 : 1 ratio), linezolid, clindamycin, cefepime, azithromycin, erythromycin, trimethoprim, sulfametoxazole, and vancomycin. Using qPCR targeting the *lytA* gene, we detected three specimens (Specimen IDs 371016106, 670710542, and 371017224) to be positive for *S. pneumoniae* with Ct values of 19, 26, and 34, respectively. Specimen IDs 670710542 and 371017224 were found to be negative through direct microscopy and culture (Table 2).

**Fig. 1.**
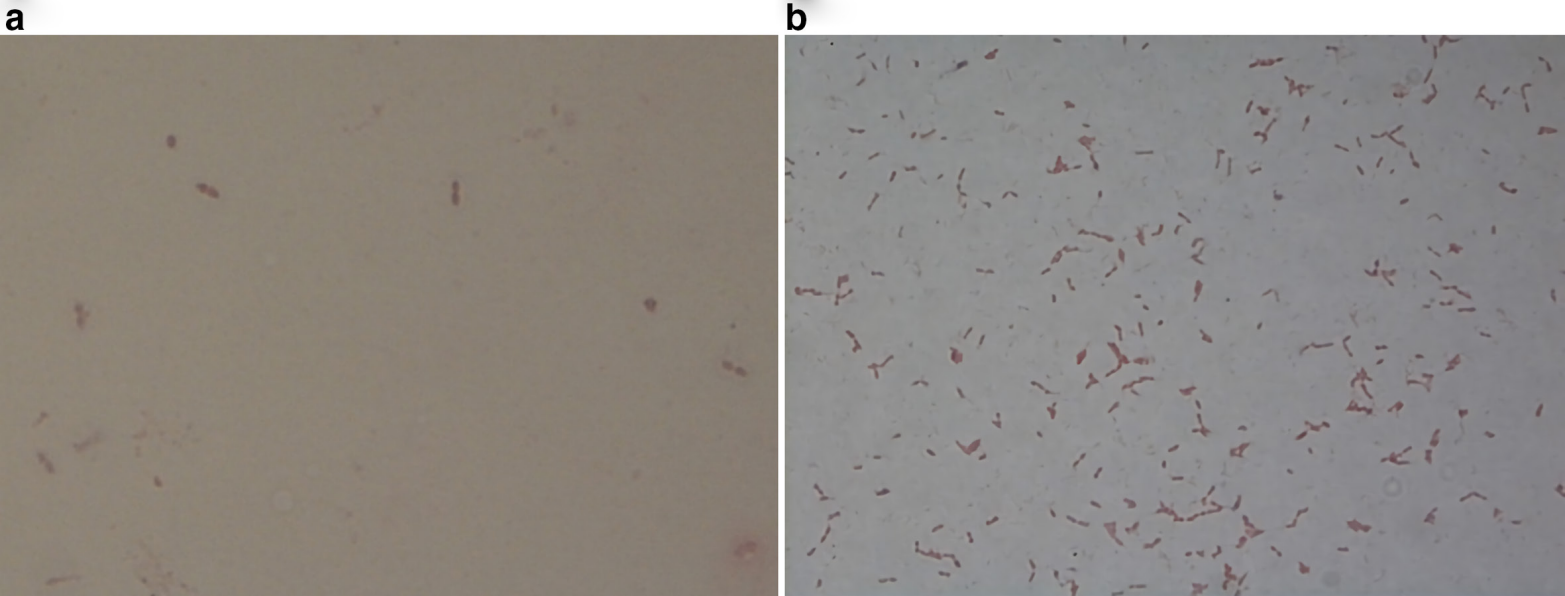
Gram-staining of a cerebrospinal fluid specimen (Specimen ID: 371016106) showing *Streptococcus pneumoniae *, a Gram-positive lanceolate-shaped diplococci, before (A) and after culture process (B), under a light microscope (1000×).

**Table 2.** Profile of CNS infection patients and specimens with positive for *Streptococcus pneumoniae
*

Variable	Specimen ID		
	371016106	670710542	371017224
Source	Emergency Department	Ward	Emergency Department
Diagnosis/clinical information	Bacterial meningitis	Tuberculous meningitis	Tuberculous meningoensephalitis
Symptoms	Fever, shortness of breath	Fever, headache	Fever, shortness of breath
Sign	Seizure, decreased consciousness, stiff neck, positive for Brudzinski test	Decreased consciousness	No neurologic sign
Antibiotic use prior to lumbal puncture	Yes	Yes	Yes
Specimen data:			
Appearance	Cloudy, yellow	Clear, colourless	Cloudy, yellow
Leukocyte count (µl^–1^)	582	76	245
PMN (%)	60	10	35
Protein (mg dl^–1^)	215	60	250
Glucose (mg dl^–1^)	11	57	43
Ratio of CSF glucose level to serum glucose level	0.1	0.6	No data
Laboratory findings:			
Culture positive for *Streptococcus pneumoniae *	Yes	No	No
Ct value of *lyt*A PCR	19	26	34
MALDI (score value)	* Streptococcus pneumoniae* (2.145)	No Test	No Test
Serotyping	6B	No Test	No Test

In addition, other microbial colonies in the culture were identified using MALDI-TOF mass spectrometry and conventional assays following routine laboratory analyses in the hospitals. From these CSF specimens, *Staphylococcus epidermidis* (*n*=8), *Staphylococcus saprophyticus* (*n*=3)*, Streptococcus dysgalactiae* (*n*=1), *Bacillus* sp. (*n*=5), *Enterobacter cloacae* (*n*=1), *Corynebacterium* sp. (*n*=1), and *Acinetobacter baumannii* (*n*=2) were isolated. Colonies on the blood agar plate from two other specimens were identified as *Cryptococcus* sp., which were confirmed by India ink examination (routine CSF analysis results; data not shown). We also observed from routine laboratory test results that 65 of the 147 CSF specimens (44.2%) were directly examined for acid-fast bacilli (* Mycobacterium tuberculosis *) either through Ziehl-Neelsen staining, culture, or real time PCR. Tuberculous meningitis was confirmed for 11 specimens (16.9%), i.e. one positive culture and 10 positive real time PCR results (data not shown)

One specimen (Specimen ID 371016106) in this study that was positive for *S. pneumoniae* following direct microscopic examination and culture belonged to a patient who was admitted to the emergency department with a major complaint of asphyxia in the last 6 h prior to admission (Table 2). In addition to shortness of breath, the patient had experienced fever and cough the previous week. During treatment at the emergency department, the patient experienced seizures three times and lost consciousness. Physical examination revealed that the patient had a stiff neck and a positive Brudzinski sign. Blood culture examination showed bacteremia and was positive for *S. pneumoniae* (data not shown). CSF was cloudy and yellow in appearance with a cell count of 582 µl^–1^ (60 % polymorphonuclear), glucose level of 11 mg dl^–1^ (serum glucose level was 98 mg dl^–1^), and protein content of 215 mg dl^–1^, which was typical for bacterial meningitis. Accordingly, the patient was administered 300 mg ceftriaxone every 12 h intravenously for 14 days. The first administration as an empirical therapy initiated approximatly 3 h before the CSF specimen was collected.

The other two specimens (Specimen IDs 670710542 and 371017224) were positive for *S. pneumoniae* following qPCR analyses (Ct values 26 and 34, respectively), but negative for direct microscopic and culture. Specimen ID 670710542 was collected from a patient, who had lost consciousness and was clinically diagnosed with tuberculous meningitis. The CSF of this patient was clear and colourless with a cell count of 76 µl^–1^ (90 % mononuclear), glucose level of 57 mg dl^–1^ (serum glucose level was 93 mg dl^–1^), and protein content of 60 mg dl^–1^. *M. tuberculosis* culture and PCR analysis showed negative results (Table 2). Specimen ID 371017224 was collected from a patient treated in the emergency department, who experienced shortness of breath and fever; subsequently, the patient was clinically diagnosed with tuberculous meningoencephalitis (Table 2). The CSF of this patient was cloudy and yellow in appearance, with a cell count of 245 µl^–1^ (65 % mononuclear), glucose level of 43 mg dl^–1^ (serum glucose level was not examined), and protein content of 250 mg dl^–1^. *M. tuberculosis* culture and Ziehl-Neelsen staining showed negative results (Table 2). Both patients had been administered ceftriaxone for more than 72 h before the CSF specimens were collected.

To the best of our knowledge, this is the first study to detect *S. pneumoniae* in CSF using conventional (Gram-staining, culture) and molecular methods from clinically diagnosed CNS infection patients in Jakarta, Indonesia. Overall, three cases of pneumococcal meningitis were identified; one was detected through culture, Gram-staining, and qPCR analyses, while the remaining cases were only detected by qPCR. A study from Bangladesh reported a higher proportion of pneumococcal meningitis, 25 of 401 (6.2 %) [19]. However, compared to invasive pneumococcal diseases (IPD) in general, our finding is similar to that of a hospital-based study conducted in Jakarta, Indonesia which reported that among 205 children hospitalized with invasive pneumococcal diseases (pneumoniae, sepsis, and meningitis), *S. pneumoniae* was identified from blood specimen as an etiological agent of the disease in only one child [20]. This serotype 6B (ST5661) finding in a patient with CNS infection shows that serotype 6B is invasive which is covered by pneumococcal conjugate vaccine (PCV). This finding showed that an invasive serotype of *S. pneumoniae* is still circulating and causing disease in Indonesia.

The sensitivity of Gram-staining and culture from CSF specimens depends on the bacterial load, meningeal pathogen type, and initiation of antibiotic therapy before lumbar puncture [21, 22]. The use of antibiotics prior to CSF collection may contribute to the reduced sensitivity of these conventional methods results, since approximately 60 % of the subjects in this study had received prior antibiotic therapy. In 25–50 % of children with suspected bacterial meningitis, the CSF is obtained after receiving antibiotics [23]. However, the CSF becomes sterile from pneumococci within at least 4 h of initiation of appropriate antibiotic therapy, and this takes even a shorter duration of time for meningococci [24]. Meanwhile, pleocytosis with a predominance of polymorphonuclear cells, elevated protein levels, and a reduced concentration of CSF glucose concentration often persist for several days [23]. Children under two years of age comprise one of the highest risk groups for IPD, in addition to ethnicity, geographic location, crowded living conditions, lower socioeconomic status, and concomitant chronic illnesses [24, 25]. Pathological conditions that increase the risk of IPD are chronic liver disease, anatomic abnormalities such as skull fracture/cerebrospinal fluid leak, cochlear implant or congenital heart disease, immunosuppressive therapy, bone marrow, and solid organ transplantation, chronic diseases (pulmonary and neurological), diabetes mellitus, and renal conditions (renal insufficiency or nephrotic syndrome) [26]. A high degree of bacteremia has been shown to be a primary determinant for meningeal invasion by *S. pneumoniae *. Bacterial blood counts >10^3^ ml^–1^ results in higher incidence of meningitis than those <10^3^ ml^–1^ [27]. However, Carrol *et al*. reported that high pneumococcal DNA loads in blood and CSF are associated with fatal outcomes in children with IPD [28].

The *S. pneumoniae* serotype 6B detected in this study is one of the predominant serotypes in Indonesia [12, 29–31]. The World Health Organization (WHO) has recommended vaccination with seven-valent pneumococcal conjugate vaccine (PCV7) and 13-valent pneumococcal conjugate vaccine (PCV13) [32]. However, pneumococcal vaccination is not part of the routine national childhood immunization program in Indonesia. Since 2017, PCV13 has been introduced in selected regions of Indonesia namely Lombok Island, West Nusa Tenggara and Bangka Belitung Island. Seven previous studies have reported reductions in vaccine-type pneumococcal meningitis incidence in vaccine-eligible children in the post-vaccination period, compared to the pre-vaccination period, ranging from 59.2 % in the United States in one year, to 100 % in Belgium four years after vaccine introduction [33]. Furthermore, the incidence of pneumococcal meningitis has been found to decline significantly in countries where PCV13 (or PCV10) vaccination has been widely implemented [7].

The limitation of this study is that we used remaining CSF specimens obtained from a hospital because the CSF sample were required for other routine hospital tests. Additionally, some CSF samples were stored temporarily at −20 °C before being processed in the laboratory. Although we detected *S *. pneumoniae using qPCR, these limitations may have slightly affected the success rate of bacterial isolation using blood agar media.

In conclusion, *S. pneumoniae* of serotype 6B (ST5661) was isolated in one patient with CNS infection in Jakarta, Indonesia. This finding showed invasive serotypes of *S. pneumoniae* are still circulating in Indonesia and causing diseases. This study suggests that CSF should be collected prior to antibiotic treatment to increase the rate of isolation. A further study about the effect of antibiotic treatment should be done to have better understanding of antibiotic treatment affecting the success rate of *S. pneumoniae* isolation.
